# Characteristics and neurological survival following intraoperative cardiac arrest in a Swiss University Hospital: a 7-year retrospective observational cohort study

**DOI:** 10.3389/fmed.2023.1198078

**Published:** 2023-06-15

**Authors:** Alexander Fuchs, Lea Franzmeier, Marie Cheseaux-Carrupt, Martina Kaempfer, Nicola Disma, Urs Pietsch, Markus Huber, Thomas Riva, Robert Greif

**Affiliations:** ^1^Department of Anaesthesiology and Pain Medicine, Bern University Hospital, Inselspital, University of Bern, Bern, Switzerland; ^2^Unit for Research in Anaesthesia, Department of Paediatric Anaesthesia, IRCCS Istituto Giannina Gaslini, Genova, Italy; ^3^Department of Emergency Medicine, Bern University Hospital, Inselspital, University of Bern, Bern, Switzerland; ^4^Department of Anaesthesiology and Intensive Care Medicine, Cantonal Hospital St. Gallen, St. Gallen, Switzerland; ^5^University of Bern, Bern, Switzerland; ^6^School of Medicine, Sigmund Freud University Vienna, Vienna, Austria; ^7^ERC Research Net, Niel, Belgium

**Keywords:** anesthesia, cardiac arrest, cardiopulonary resuscitation, perioperative care (intraoperative care), ROSC (return of spontaneous circulation), functional outcomes, health-related quality of life

## Abstract

**Introduction:**

Little is known about intraoperative cardiac arrest during anesthesia care. In particular, data on characteristics of cardiac arrest and neurological survival are scarce.

**Methods:**

We conducted a single-center retrospective observational study evaluating anesthetic procedures from January 2015 until December 2021. We included patients with an intraoperative cardiac arrest and excluded cardiac arrest outside of the operating room. The primary outcome was the return of spontaneous circulation (ROSC). Secondary outcomes were sustained ROSC over 20 min, 30-day survival, and favorable neurological outcome according to Clinical Performance Category (CPC) 1 and 2.

**Results:**

We screened 228,712 anesthetic procedures, 195 of which met inclusion criteria and were analyzed. The incidence of intraoperative cardiac arrest was 90 (CI 95% 78–103) in 100,000 procedures. The median age was 70.5 [60.0; 79.4] years, and two-thirds of patients (*n* = 135; 69.2%) were male. Most of these patients with cardiac arrest had ASA physical status IV (*n* = 83; 42.6%) or V (*n* = 47; 24.1%). Cardiac arrest occurred more frequently (*n* = 104; 53.1%) during emergency procedures than elective ones (*n* = 92; 46.9%). Initial rhythm was pre-dominantly non-shockable with pulseless electrical activity mostly. Most patients (*n* = 163/195, 83.6%; CI 95 77.6–88.5%) had at least one instance of ROSC. Sustained ROSC over 20 min was achieved in most patients with ROSC (*n* = 147/163; 90.2%). Of the 163 patients with ROSC, 111 (68.1%, CI 95 60.4–75.2%) remained alive after 30 days, and most (*n* = 90/111; 84.9%) had favorable neurological survival (CPC 1 and 2).

**Conclusion:**

Intraoperative cardiac arrest is rare but is more likely in older patients, patients with ASA physical status ≥IV, cardiac and vascular surgery, and emergency procedures. Patients often present with pulseless electrical activity as the initial rhythm. ROSC can be achieved in most patients. Over half of the patients are alive after 30 days, most with favorable neurological outcomes, if treated immediately.

## 1. Introduction

Cardiac arrest is one of the leading causes of death in Europe (1). Patients with cardiac arrest must be treated immediately with basic life support to minimize no-flow time. Basic life support includes chest compressions with ventilation of the lungs, defined as cardiopulmonary resuscitation (CPR), and early defibrillation (2, 3). Early detection of cardiac arrest, high-quality CPR, and prompt defibrillation are crucial for patients’ survival with favorable neurological outcomes (4, 5). To ensure a favorable outcome, reversible causes of cardiac arrest—referred to by the mnemonic H’s and T’s—must be diagnosed and treated (4). Intraoperative cardiac arrest is a unique form of in-hospital cardiac arrest (IHCA) (6–8) and is feared by patients undergoing anesthesia (9). The limited data that exist suggest that the incidence of perioperative cardiac arrest is between 0.5 and 3 per 10,000 procedures for adult patients (10–13) and between 0.5 and 10 per 10,000 procedures for pediatric patients (14–18).

Patients undergoing anesthesia are considered highly monitored, which may contribute to early detection of cardiac arrest triggering the start of the chain of survival (6, 19). Furthermore, the personnel and equipment needed to provide advanced life support are assumed to be on site already, further accelerating proper resuscitative efforts. On the other hand, there has been a demographic change in patients undergoing anesthesia over the last decades. Nowadays, patients are older and present with a higher BMI and American Society of Anesthesiologists (ASA) physical status score (20), which contributes to a higher baseline risk for cardiac arrest (13).

A registry study with data collected from 2008 until 2012, including more than 1.8 million non-cardiac operations, identified ASA physical status, anesthesia technique, case urgency, type of surgery, and systemic inflammatory response syndrome (SIRS)/sepsis as the strongest predictors of intraoperative cardiac arrest (10). The in-hospital mortality after intraoperative cardiac arrest was reported to be around 35%, and 30-day mortality was up to 71% (21, 22). Some reports suggested that incidence is negatively associated with the higher resource areas (13, 23).

Data on the characteristics of intraoperative cardiac arrest are scarce. Furthermore, most published studies focused only on survival and did not investigate neurological outcome. Therefore, the sequela of intraoperative cardiac arrest for patients regarding neurological outcomes and health-related quality of life is underreported. Thus, we conducted our study to bridge this knowledge gap.

## 2. Materials and methods

### 2.1. Ethics committee approval and trial registration

The study protocol was approved by the responsible Cantonal Ethics Committee of Bern (BASEC 2021-02330), and the trial was registered with ClinicalTrials.gov (NCT05316779). For the retrospective part of the study, existing general consent was checked, and for the telephone interview, informed consent was obtained.

### 2.2. Setting

The Bern University Hospital is one of the largest academic university-affiliated hospitals in Switzerland, with emergency departments for children and adults, and is a certified cardiac arrest center (24). The Department of Anaesthesiology and Pain Medicine oversees around 32,000 procedures annually, including about 23,360 (73%) electives and 8,640 (27%) emergency procedures (25). Patients scheduled for elective surgery have a visit to the pre-anesthetic clinic around 2–8 weeks before the surgery. This aims to assess the individual perioperative risk for morbidity and mortality regarding patient and surgery-related factors. Therefore, the anesthesiologist uses the ASA physical status, a classification system considering the patients’ medical co-morbidities within five categories. In brief, a patient with ASA physical status 1 is healthy and ASA physical status 5 is moribund (26). The higher the category, the higher the peri-operative risk for morbidity and mortality (27). Individual patient-centered optimization can be undertaken during and after the pre-anesthetic visit by following departmental standard operating procedures and current guidelines (28).

Specialized pediatric anesthesiologists provide anesthesia for children <16 years of age in the Bern Children’s Hospital operating rooms. Post-resuscitation care is provided by specialized pediatric critical care physicians in the dedicated pediatric intensive care unit. Adolescents over 16 years are treated in the adults’ hospital areas. All clinical staff undergo a mandatory annual one-day European Resuscitation Council Advanced Life Support refresher course in groups of 6 persons to ensure that CPR competencies conform to current resuscitation guidelines. Furthermore, each staff member has a one-day interprofessional and interdisciplinary *in-situ* high-fidelity simulation training in the operating room, followed by video debriefing.

### 2.3. Participants

We included all cardiac arrest patients treated in the 7 years between January 1, 2015 and December 31, 2021 in the operating room under anesthesia care after the departmental anesthesia sign-in (25). We excluded patients with cardiac arrest outside the operating room (i.e., cardiac catheter laboratory, wards, intensive care units, emergency room, or other locations in the hospital), patients admitted to the operating room under ongoing CPR, and procedures with planned extracorporeal circulation or cardiopulmonary bypass after cannulation.

### 2.4. Procedures and measures

We conducted a two-phased single-center retrospective observational study. In the first phase, between April 14 and May 31, 2022, we screened the departmental anesthesia information system for patients with cardiac arrest. We defined cardiac arrest as at least five chest compressions or one defibrillation having been delivered to the patient, according to the definition of the 7th National Audit Project (29). Data were extracted into the departmental Research Electronic Data Capture (REDCap, Vanderbilt University, United States) using the Utstein-style template for in-hospital cardiac arrest (30). The data included patients’ demographics (sex, age, weight, and height), medical data (ASA physical status, cardiac and vascular surgery vs. non-cardiac surgery), and emergency category (elective vs. emergency procedure). In a second step, between May 1 and June 30, 2022, we invited all survivors to a telephone interview to assess their neurological status. We followed the applicable strengthening the reporting of observational studies in epidemiology (STROBE) guidelines (31).

#### 2.4.1. Primary outcome

The primary endpoint was the return of spontaneous circulation (ROSC) over 1 min at any time during cardiopulmonary resuscitation.

#### 2.4.2. Secondary outcomes

Secondary outcomes were sustained ROSC over 20 min, 30-day survival with favorable neurological outcome, and 3 months of survival. Neurological outcome was assessed with the cerebral performance category (CPC) (32). The CPC has a five-point scale, with lower numbers indicating better neurological outcomes. Favorable neurological survival was defined as CPC 1 and 2. Patients’ health-related quality of life was assessed with the Short Form 12 (SF-12), which has two core dimensions, the Mental component summary and the Physical component summary, covered by 12 questions, each calculated on a scale of 0–100 (33). The score is age-dependent, with higher values corresponding to better health-related quality of life. The telephone interview captured the SF-12 and the CPC for three different time points: (I) at 30 days after cardiac arrest, (II) at three months after cardiac arrest, and (III) at the time point of the telephone interview. If the telephone interview was not possible, the 30-day CPC was retrospectively determined based on documentation from the electronic medical record.

### 2.5. Statistical analysis

Categorical variables were given in absolute numbers and percentages. Continuous variables were presented as means ± standard deviation (SD) and skewed data with median and interquartile range [IQR]. Student’s *t*-tests were used to compare continuous, normally distributed data, and Mann–Whitney or Kruskal–Wallis tests for skewed data. Categorical variables were compared with chi-squared tests or Fisher’s exact tests. As exploratory analyses, univariable odds ratios were derived for a pre-selected number of variables employing logistic regression for the two binary outcomes ROSC (yes vs. no) and CPC (categories 1–2 vs. 3–5). A survival analysis using Kaplan–Meier estimates was computed for the entire cohort. We computed univariable, between-group significance tests in terms of survival times using a log-rank test concerning the type of surgery (cardio and vascular vs. non-cardiac), urgency (emergency vs. non-emergency) and the type of the initial cardiac rhythm (shockable vs. non-shockable). The significance level of probability was defined as ≤0.05. All calculations were performed with the R statistical software (34).

## 3. Results

**Figure 1:**
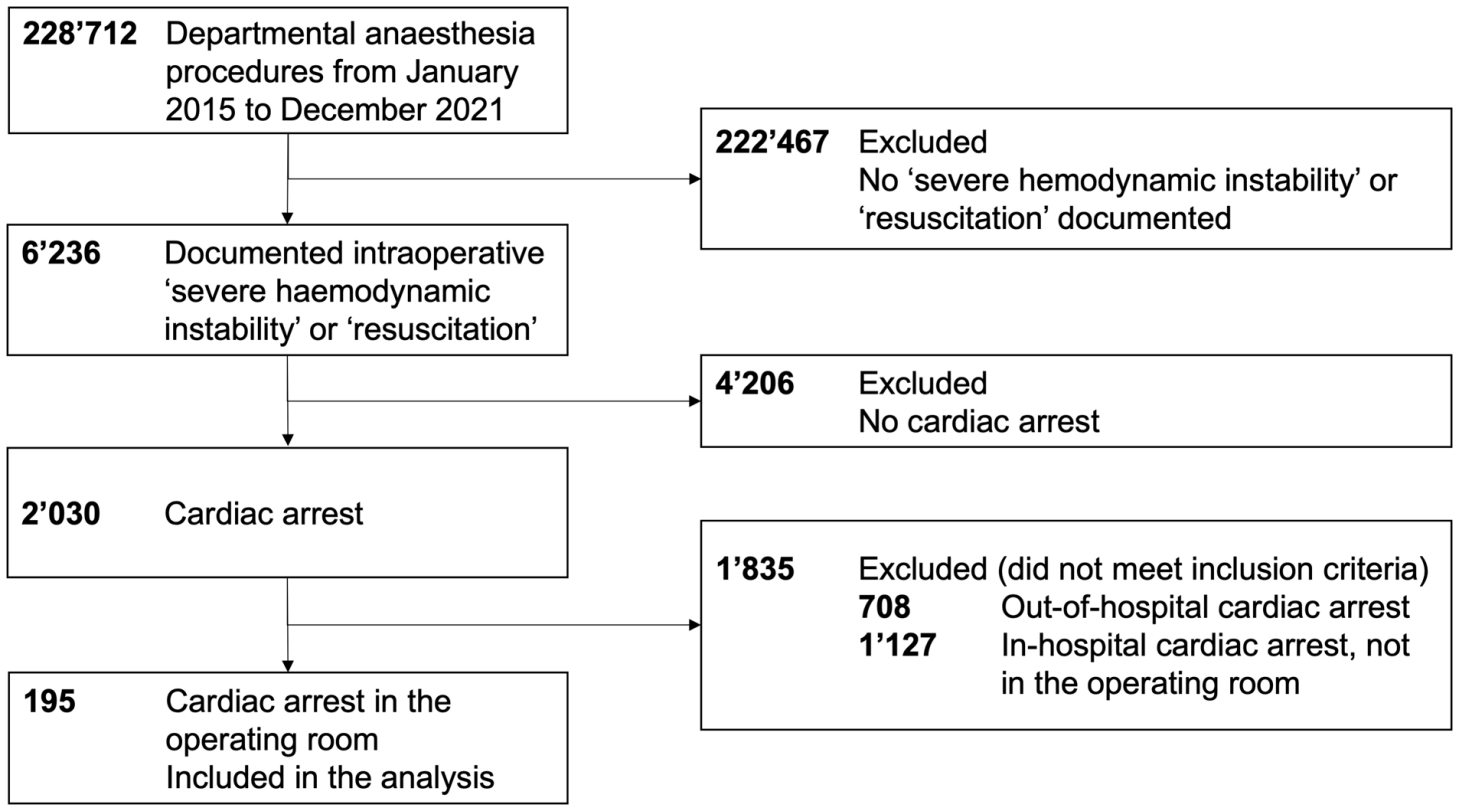
Study flowchart.

We screened 228,712 departmental anesthesia procedures, 6,245 of which were labeled with the header “severe cardiopulmonary instability” or “resuscitation” in the electronic medical record. Last, we included 195 patients with intraoperative cardiac arrest in this analysis, as displayed in Figure 1.

Patients’ baseline characteristics are summarized in Table 1. The median age was 70.5 [60.0; 79.4] years, and two-thirds of patients (*n* = 135; 69.2%) were male. Most patients had an ASA physical status ≥IV (IV; *n* = 83; 42.6% and V, *n* = 47; 24.1%). Cardiac arrest occurred more frequently (*n* = 103; 53.1%) during emergency procedures than elective ones (*n* = 92; 46.9%). The responsible surgeon classified almost one-third of the procedures (32.3%; *n* = 63) as immediate urgent intervention (Supplemental Tables S1, S2). The overall annual incidence of intraoperative cardiac arrest was 90 (CI 95% 78–103) in 100,000 procedures, with 24 (CI 95% 10–50) in children and 100 (CI 95% 86–115) in adults, respectively. The estimated annual incidence of intraoperative cardiac arrest was 58 (CI 95% 47–71) in 100,000 for elective and 175 (CI 95% 143–213) in 100,000 for emergency procedures.

**Table 1:** Baseline characteristics of the patients with intraoperative cardiac arrest.

	All patients	Adults (≥16 yrs)	Children (<16 yrs)	*p*	*N*
	*N = 195*	*N = 188*	*N = 7*		
Age (yrs)	70.5 [60.0;79.4]	71.3 [61.4;79.6]	1.36 [0.14;8.99]	–	195
Sex (female)	60/195 (30.8%)	59/188 (31.4%)	1/7 (14.3%)	0.44	195
Height (cm)	170 [163;178]	171 [164;178]	74.0 [58.0;156]	–	169
Weight (kg)	75.5 [64.2;89.8]	76.0 [65.0;90.0]	9.00 [3.50;31.0]	–	178
BMI (kg.m^−2^)	26.5 (5.59)	26.8 (5.31)	16.6 (5.95)	–	168
ASA physical status				0.40	195
I	1 (0.51%)	1 (0.53%)	0 (0.00%)		
II	14 (7.18%)	13 (6.91%)	1 (14.3%)		
III	50 (25.6%)	48 (25.5%)	2 (28.6%)		
IV	83 (42.6%)	79 (42.0%)	4 (57.1%)		
V	47 (24.1%)	47 (25.0%)	0 (0.00%)		
Pre-existing illness (Yes)	181/195 (94.3%)	175/188 (94.6%)	6/7 (85.7%)	0.34	192
Pre-existing illness category^1^					
Cardiovascular	149/195 (76.4%)	145/188 (77.1%)	4/7 (57.1%)	0.36	195
Pulmonary	73/195 (37.4%)	69/188 (36.7%)	4/7 (57.1%)	0.43	195
Neurological	46/195 (23.6%)	46/188 (24.5%)	0/7 (0%)	0.20	195
Renal	76/195 (39.0%)	76/188 (40.4%)	0/7 (0%)	0.044	195
Cancer	49/195 (25.1%)	49/188 (26.1%)	0/7 (0%)	0.20	195
Pregnancy	1/195 (0.5%)	1/188 (0.5%)	0/7 (0%)	>0.99	195
Other^2^	33/195 (16.9%)	33/188 (17.6%)	0/7 (0%)	0.61	195
Pre-existing condition^1^					
No	96/195 (49.2%)	90/188 (47.9%)	6/7 (85.7%)	0.06	195
Yes					
Sepsis	17/195 (8.7%)	16/188 (8.5%)	1/7 (14.3%)	0.48	195
Hypotension	31/195 (15.9%)	31/188 (16.5%)	0/7 (0%)	0.60	195
Metastatic/hematological malignancy	18/195 (9.2%)	18/188 (9.6%)	0/7 (0%)	>0.99	195
Hepatic/renal insufficiency	72/195 (36.9%)	72/188 (38.3%)	0/7 (0%)	0.048	195
Urgency of the procedure				0.69	195
Emergency	103/195 (53.8%)	101/188 (53.7%)	2/7 (28.6%)		
Immediate	63/195 (32.3%)	62/188 (33.0%)	1/7 (14.3%)		
Within 1–6 h	28/195 (14.4%)	27/188 (14.4%)	1/7 (14.3%)		
Within 6–12 h	5/195 (2.6%)	5/188 (2.7%)	0/7 (0%)		
Within 24 h	7/195 (3.6%)	7/188 (3.7%)	0/7 (0%)		
Elective	92/195 (47.2%)	87/188 (46.3%)	5/7 (71.4%)		
Anaesthesia procedure				>0.99	195
Regional	3/195 (1.5%)	3/188 (1.6%)	0/7 (0%)		
General and combined anaesthesia	180/195 (92.3%)	173/188 (92.0%)	7/7 (100%)		
Monitored anaesthesia care	12/195 (6.2%)	12/188 (6.4%)	0/7 (0%)		
Surgical intervention				0.60	195
Open	165/195 (84.6%)	158/188 (84.0%)	7/7 (100%)		
Endovascular	30/195 (15.4%)	30/188 (16.0%)	0/7 (0.00%)		
Type of surgery				0.71	195
Non-cardiac	124/195 (63.6%)	120/188 (63.8%)	4/7 (57.1%)		
Cardiac and vascular	71/195 (36.4%)	68/188 (36.2%)	3/7 (42.9%)		

ASA, American Society of Anesthesiologist; BMI, Body Mass Index.

1 A patient could have more than one pre-existing illness or condition.

2 Contained but not limited metabolic, psychiatric, liver, and hematological disorders.

### 3.1. Intraoperative cardiac arrest

Cardiac arrest-related data are summarized in Table 2. The median time to cardiac arrest after the start of anesthesia was 109 [41.0; 224.0] minutes. Initial rhythm was pre-dominantly non-shockable and mostly pulseless electrical activity (PEA). The median duration of CPR until ROSC was 5.0 [2.0; 14.0] minutes. One-third of the patients (*n* = 64; 32.8%) received one or more defibrillations during CPR. Univariable (unadjusted) odds ratios for the outcome ROSC were lower for patients with ASA physical status IV-V (OR 0.11; CI 95% 0.02–0.37; *p* = 0.003) and cardiac and vascular surgery (OR 0.37; CI 95% 0.17–0.81; *p* = 0.013), as summarized in Table 3. Longer CPR duration in minutes also had a lower odds ratio for ROSC (OR 0.94; CI 95% 0.92–0.97; *p* < 0.001), but was only an effect modulator.

**Table 2:** Intraoperative cardiac arrest related data.

	All patients	Adults (=16 yrs)	Children (<16 yrs)	*p*	*N*
	*N = 195*	*N = 188*	*N = 7*		
Time of day				>0.99	195
Daytime (7:00–17:00 h)	133 (68.2%)	128 (68.1%)	5 (71.4%)		
Nighttime	62 (31.8%)	60 (31.9%)	2 (28.6%)		
Time to cardiac arrest after start of anesthesia (min)	109 [41.0;224]	113 [41.5;226]	27.5 [12.5;87.5]	0.088	193
Initial rhythm during cardiac arrest				0.043	194
*Shockable*	45 (23.2%)	44 (23.5%)	1 (14.3%)		
Ventricular fibrillation	28 (14.4%)	27 (14.4%)	1 (14.3%)		
Pulseless Ventricular Tachycardia	15 (7.7%)	15 (8.02%)	0 (0%)		
Shockable, not further specified	2 (1.03%)	2 (1.07%)	0 (0%)		
*Non-shockable*	149 (76.8%)	143 (76.5%)	6 (85.7%)		
Pulseless electrical activity	88 (45.4%)	86 (46.0%)	2 (28.6%)		
Asystole	24 (12.4%)	24 (12.8%)	0 (0%)		
Bradycardia	2 (1.0%)	0 (0%)	2 (28.6%)		
Non-shockable, not further specified	32 (16.5%)	30 (16.0%)	2 (28.6%)		
Reasons for cardiac arrest					
Tamponade (cardiac)	14 (7.18%)	14 (7.45%)	0 (0%)	>0.99	195
Intoxication	1 (0.51%)	1 (0.53%)	0 (0%)	>0.99	195
Tension pneumothorax	1 (0.51%)	0 (0%)	1 (14.3%)	0.036	195
Hypoxia	12 (6.15%)	11 (5.85%)	1 (14.3%)	0.364	195
Hypovolemia	54 (27.7%)	54 (28.7%)	0 (0%)	0.193	195
Hypothermia	0 (0%)	0 (0%)	0 (0%)	.	195
Hypo-hyperpotassemia	8 (4.10%)	8 (4.26%)	0 (0%)	>0.99	195
Hypoglycemia	0 (0%)	0 (0%)	0 (0%)	.	195
Thrombosis (pulmonary)	5 (2.56%)	5 (2.66%)	0 (0%)	>0.99	195
Thrombosis (coronary)	4 (2.05%)	3 (1.60%)	1 (14.3%)	0.137	195
Hydrogen ion (acidosis)	6 (3.08%)	6 (3.19%)	0 (0%)	>0.99	195
Unknown	47 (24.1%)	46 (24.5%)	1 (14.3%)	>0.99	195
Other	73 (37.4%)	68 (36.2%)	5 (71.4%)	0.105	195
Duration CPR until ROSC (min)	5.00 [2.0;14.0]	5.00 [2.0;14.0]	17.0 [5.5;33.0]	0.113	192
Defibrillation during CPR	64 (32.8%)	62 (33.0%)	2 (28.6%)	>0.99	195
Number of shocks given					
1	59 (30.3%)	58 (30.9%)	1 (14.3%)	0.677	195
2	33 (16.9%)	33 (17.6%)	0 (0%)	0.605	195
3	17 (8.72%)	17 (9.04%)	0 (0%)	>0.99	195
4	8 (4.10%)	8 (4.26%)	0 (0%)	>0.99	195
5	4 (2.05%)	4 (2.13%)	0 (0%)	>0.99	195
6	3 (1.54%)	3 (1.60%)	0 (0%)	>0.99	195
7	4 (2.05%)	3 (1.60%)	1 (14.3%)	0.137	195
Medication peri-arrest					
Epinephrine	167 (85.6%)	161 (85.6%)	6 (85.7%)	>0.99	195
Norepinephrine	116 (59.5%)	114 (60.6%)	2 (28.6%)	0.122	195
Amiodarone	24 (12.3%)	23 (12.2%)	1 (14.3%)	>0.99	195
Lidocaine	4 (2.05%)	4 (2.13%)	0 (0%)	>0.99	195
Vasopressin	13 (6.67%)	13 (6.91%)	0 (0%)	>0.99	195
Atropine	21 (10.8%)	17 (9.04%)	4 (57.1%)	0.003	195
Bicarbonate	27 (13.8%)	25 (13.3%)	2 (28.6%)	0.250	195
Calcium	43 (22.1%)	43 (22.9%)	0 (0%)	0.351	195
Magnesium	13 (6.67%)	13 (6.91%)	0 (0%)	>0.99	195
Additional actions during CPR					
(Arterial) Blood test	66 (33.8%)	62 (33.0%)	4 (57.1%)	0.230	195
Pericardiocentesis	15 (7.69%)	14 (7.45%)	1 (14.3%)	0.434	195
Chest tube	1 (0.51%)	0 (0%)	1 (14.3%)	0.036	195
Transesophageal Echocardiography	69 (35.4%)	66 (35.1%)	3 (42.9%)	0.700	195
Aortic clamping	19 (9.74%)	19 (10.1%)	0 (0%)	>0.99	195
RBC transfusion	74 (37.9%)	72 (38.3%)	2 (28.6%)	0.711	195
FFP transfusion	18 (9.23%)	18 (9.57%)	0 (0%)	>0.99	195

CPR, cardiopulmonary resuscitation; ROSC, return of spontaneous circulation; RBC, red blood cell; FFP, fresh frozen plasma.

**Table 3:** Univariable (unadjusted) odds ratios for the outcomes (A) return of spontaneous circulation (ROSC) and (B) 30-day Clinical Performance Category (CPC) 1 and 2 indicating favorable neurological survival.

Characteristic	ROSC (Yes vs. No)				30-day CPC (1–2 vs. 3–5)			
	*N*	OR	CI 95%	*p*	*N*	OR	CI 95%	*p*
Age (years)	195	1.01	0.99, 1.03	0.5	111	0.99	0.96, 1.02	0.6
ASA physical status	195				111			
ASA I-III		–	–			–	–	
ASA IV-V		0.11	0.02, 0.37	0.003		0.34	0.09, 1.06	0.079
Cardiac and vascular surgery	195				111			
No		–	–			–	–	
Yes		0.37	0.17, 0.81	0.013		0.78	0.27, 2.37	0.7
Initial rhythm during cardiac arrest	162				90			
Non-shockable		–	–			–	–	
Shockable		0.48	0.19, 1.19	0.10		0.72	0.21, 2.88	0.6
Duration of CPR (min)	192	0.94	0.92, 0.97	<0.001	109	0.97	0.93, 1.02	0.2
Defibrillation during CPR	195				111			
No		–	–			–	–	
Yes		0.67	0.31, 1.48	0.3		1.07	0.34, 4.11	>0.9

ASA, American Society of Anesthesiologists; CI, confidence interval; CPC, clinical performance category; CPR, cardiopulmonary resuscitation; OR, odds ratio.

### 3.2. Survival and neurological outcomes

**Figure 2:**
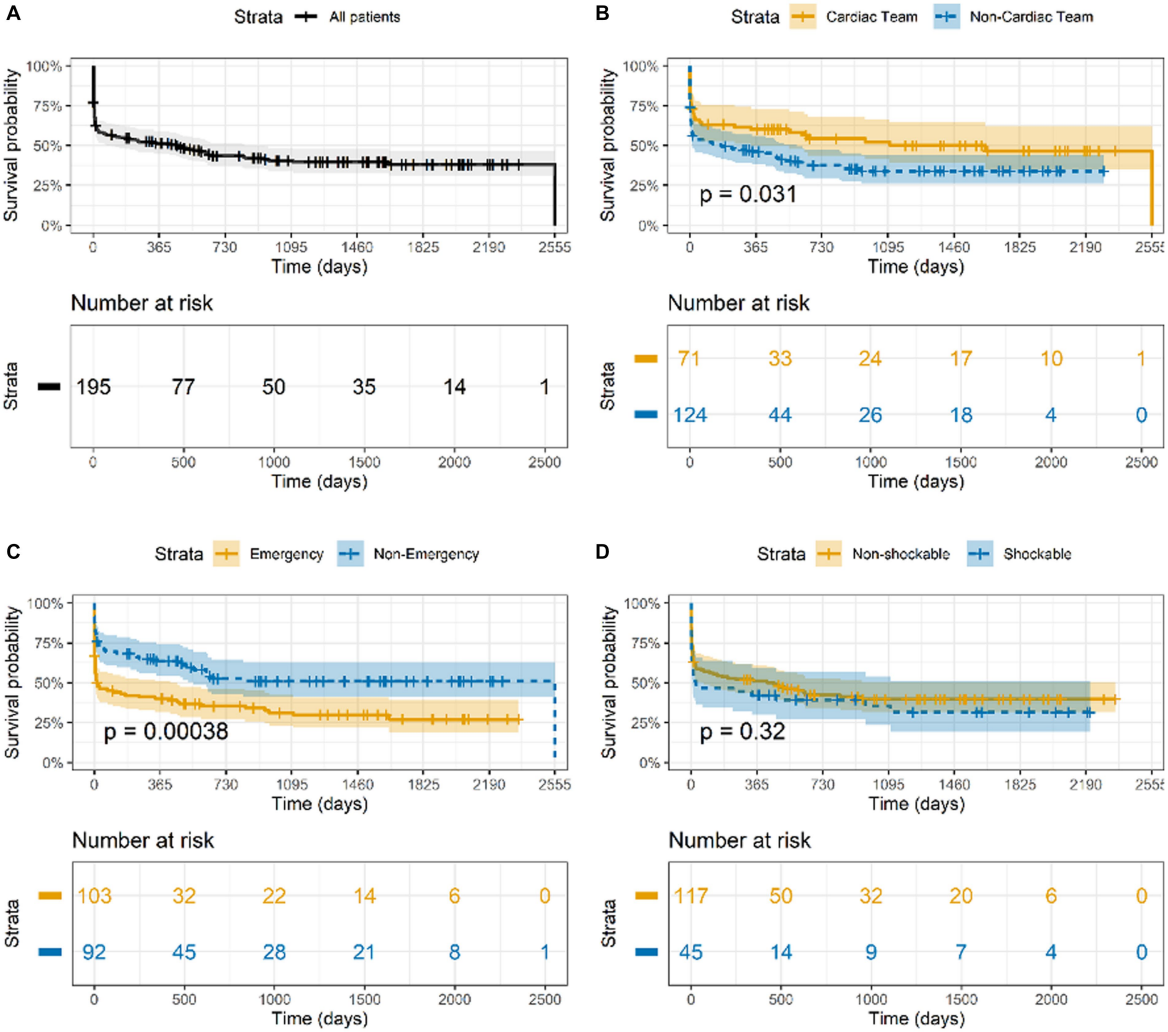
Kaplan–Meier survival estimates for **(A)** all patients, **(B)** emergency vs. elective procedures, **(C)** cardiac and vascular vs. non-cardiac surgery, and **(D)** shockable vs. non-shockable first rhythm during cardiac arrest.

Primary and secondary outcomes are summarized in Table 4. Most patients (*n* = 163/195, 83.6%; CI 95 77.6–88.5%) had ROSC at least once, while the others died (*n* = 20/32; 62.5%) or received extracorporeal cardiopulmonary resuscitation (ECPR) (*n* = 12/32; 37.5%). Sustained ROSC over 20 min was achieved in most patients with ROSC (*n* = 147/163; 90.2%). Of the 163 patients with ROSC, 111 (68.1, 60.4–75.2%) remained alive after 30 days (Supplemental Tables S3, S4). Most (*n* = 90; 84.9%) of the patients with ROSC had favorable neurological survival (CPC 1 and 2). Long-term survival was higher for elective procedures than for emergency procedures, as displayed in Figure 2. However, we could not determine which factors produced higher odds ratios for favorable neurological survival, as summarized in Table 3.

**Table 4:** Survival and neurological follow-up of the patients with intraoperative cardiac arrest.

	All patients	Adults (≥16 yrs)	Children (<16 yrs)	*p*
Return of spontaneous circulation (ROSC)				
ROSC	163/195 (83.6, 77.6–88.5%)	158/188 (84.0, 78.0–89.0%)	5/7 (71.4, 29.0–96.3%)	0.32
Reason no ROSC				0.13
ECPR	12/32 (37.5%)	10/30 (33.3%)	2/2 (100%)	
Death	20/32 (62.5%)	20/30 (66.7%)	0/2 (0.00%)	
Death after ECPR	10/12 (83.3%)	9/10 (90.0%)	1/2 (50.0%)	0.32
Actions after ROSC				
Intubation	15/163 (9.2%)	15/158 (9.5%)	0/5 (0%)	>0.99
Pacemaker insertion	3/163 (1.8%)	3/158 (1.9%)	0/5 (0%)	>0.99
Percutaneous coronary intervention	3/163 (1.8%)	3/158 (1.9%)	0/5 (0%)	>0.99
Lysis	2/163 (1.2%)	2/158 (1.3%)	0/5 (0%)	>0.99
ECLS	9/163 (5.5%)	8/158 (5.1%)	1/5 (20.0%)	0.25
Other^1^	122/163 (74.8%)	117/158 (74.1%)	5/5 (100%)	0.33
Sustained ROSC				
Sustained ROSC over 20 min	147/163 (90.2%)	143/158 (90.5%)	4/5 (80.0%)	0.41
Condition with sustained ROSC				
Awake	10/163 (6.1%)	10/158 (6.3%)	0/5 (0%)	>0.99
Sedated	144/163 (88.3%)	139/158 (88.0%)	5/5 (100%)	>0.99
Coma	7/163 (4.3%)	7/158 (4.4%)	0/5 (0%)	>0.99
Controlled ventilation	149/163 (91.4%)	144/158 (91.1%)	5/5 (100%)	>0.99
Assisted ventilation	1/163 (0.6%)	1/158 (0.6%)	0/5 (0%)	>0.99
Spontaneous breathing	7/163 (4.3%)	7/158 (4.4%)	0/5 (0%)	>0.99
Vasopressors^2^	127/163 (77.9%)	124/158 (78.5%)	3/5 (60.0%)	0.31
Transfer after sustained ROSC				0.56
Post-anesthesia Care Unit	17/160 (10.6%)	16/155 (10.3%)	1/5 (20.0%)	
Intermediate Care Unit	18/160 (11.2%)	18/155 (11.6%)	0/5 (0.00%)	
Intensive Care Unit	122/160 (76.2%)	118/155 (76.1%)	4/5 (80.0%)	
Cardiac Catheterization Laboratory	3/160 (1.88%)	3/155 (1.9%)	0/5 (0.00%)	
Survival outcome 30 days				
Survival 30 days of those with ROSC	111/163 (68.1, 60.4–75.2%)	106/158 (67.1, 59.2–74.3%)	5/5 (100, 47.8–100%)	0.18
Survival 30 days of all patients	111/195 (56.9, 49.7–64.0%)	106/188 (56.4, 49.0–63.6%)	5/7 (71.4, 29.0–96.3%)	0.70
CPC (30 days):				–
1	–	63/106 (59.4, 49.5–68.9%)	3/5 (60.0, 14.7–94.7%)	
2	–	27/106 (25.5, 17.5–34.9%)	1/5 (20.0, 0.5–71.6%)	
3	–	16/106 (15.1, 8.9–23.4%)	1/5 (20.0, 0.5–71.6%)	
Survival outcome 3 months				
Survival 3 months *of those with ROSC*	107/163 (65.6, 57.8–72.9%)	103/158 (65.2, 57.2–72.6%)	4/5 (80.0, 28.4–99.5%)	
Survival 3 months *of all patients*	107/195 (54.9, 47.6–62.0%)	103/188 (54.8, 47.4–62.0%)	4/7 (57.1%, 18.4–90.1%)	
Participated at follow-up		39/67	3/5	
CPC (3 months):				
1	–	34/39 (87.2, 72.6–95.7%)	2/3 (66.7, 9.4–99.2%)	
2	–	4/39 (10.3, 2.9–24.2%)	1/3 (33.3, 0.8–90.6%)	
3	–	1/39 (2.6, 0.1–13.5%)	0/3 (0, 0.0–70.8%)	
12-item Short Form Survey (3 months)				
Physical component score	43.5 (40.2–46.8)	43.3 (39.9–46.8)	45.7 (14.6–76.8)	0.78
Mental component score	50.9 (47.6–54.1)	51.0 (47.6–54.4)	49.1 (30.1–68.2)	0.73

ROSC, return of spontaneous circulation; ECPR, extracorporeal cardiopulmonary resuscitation; ECLS, extracorporeal life support; CPC, clinical performance category.

1 Routine critical care investigations including blood gas test, blood tests, X-Ray, 12-lead electrocardiogram.

2 Routine post-resuscitation care to maintain hemodynamic stability, including continuous vasopressors infusion with syringe pumps (e.g., Epinephrine or Norepinephrine).

### 3.3. Follow-up data on health-related quality of life

At the time-point of the telephone interview, 72 members of the cohort were still alive, and 42 (58.3%) participated in the assessment. The majority (*n* = 41/42; 97.6%) had favorable neurological survival 3 months after the cardiac arrest. The health-related quality of life after 3 months assessed with the SF-12 showed a lower physical component score and a slightly higher mental component score than in the average Swiss population (35).

## 4. Discussion

This retrospective single-center study assessed characteristics and neurological survival following intraoperative cardiac arrest over 7 years. The overall incidence of intraoperative cardiac arrest was 80 (CI 95% 69–92) in 100,000 procedures. Pulseless electrical activity was most often the first rhythm in this cohort. Most patients had ROSC at least once over a one-minute period and sustained ROSC over 20 min. Over half of the patients had 30-day survival, most with a favorable neurological outcome (CPC 1 and 2). The health-related quality of life assessed 3 months after the cardiac arrest with the SF-12 was lower in the physical component score but higher in the mental component score than the average Swiss population.

Our study’s cardiac arrest incidences were comparable to previously reported pediatric data (10–13) but higher for adult (14–18) patients. The higher incidence in adult patients might be explained by including cardiac and vascular surgery. However, incidences vary in their classification (e.g., cardiac and vascular vs. non-cardiac surgery or elective vs. emergency procedures) and might be a problem of definition (1). Cardiac arrest in our presented cohort occurred more frequently during emergency procedures, similar to earlier reports (10). These patients are likely underdiagnosed regarding their co-morbidities and risks for cardiac arrest compared to elective patients, who can be clinically assessed in more detail in the pre-anesthetic visit and optimized if needed (28). Thus “prevention”—identified as a crucial step to avoid or indicate preparations in case of a cardiac arrest—is often limited in emergency patients (3). Furthermore, several emergency patients, especially those with highly urgent surgery, are considered to have severe trauma (e.g., polytrauma) or non-trauma (e.g., acute aortic dissection or a diagnosis of sepsis) are not expected to survive without surgery, as reflected by the high proportion of ASA physical status V.

Pulseless electrical activity was a leading non-shockable first rhythm in the presented cohort, and might be related to hypovolemic or distributive shock. Intraoperative hypovolemic or distributive shock is characterized by reduced circulating blood volume, and can be diagnosed by severe hypotension as a surrogate. While intraoperative hemorrhage might be the most significant and obvious cause during procedures, as reflected by the high number of red blood cell transfusions in this cohort, blood loss should be carefully monitored. Investigations (e.g., measurement of hemoglobin values and hemostasis) should be undertaken to identify appropriate treatment (36). Pre-existing conditions (e.g., sepsis), pre-existing illness (e.g., renal or liver failure), and pharmacological treatment (e.g., antihypertensive, antithrombotic, or anticoagulation) can contribute to intravascular hypovolemia or vasoplegia and should be anticipated early. Avoiding perioperative hemodynamic imbalance is also a key message underlined in the European Society of Cardiology guidelines to improve patients’ outcomes in non-cardiac surgery (28). Severe anaphylaxis from medication (e.g., antibiotics, neuromuscular blocking agents) or material (e.g., latex, prosthesis) used (37), or a patient’s position during the procedure (e.g., extreme reverse Trendelenburg or beach chair positions) should also be taken into account as a cause for cardiac arrest. The anesthesiologist’s task is to prevent the relative hypovolemia caused by vasodilation due to anesthesia overdose by monitoring anesthetic depth with a patient-centered electroencephalogram, and by being vigilant concerning absolute hypovolemia by treating volume loss and hypotension at an early stage.

An analysis of more than 700 episodes of pulseless electrical activity during CPR for patients with in-hospital cardiac arrest showed that it was the primary rhythm in around 60% (*n* = 423) of their included episodes (38). ROSC was recorded in over half of these episodes, with initial pulseless electrical activity (*n* = 230). However, only 9% of these patients survived until hospital discharge. In a recent registry data analysis of over 3,400 in-hospital cardiac arrest patients, an initial shockable rhythm was associated with an increased probability of 30-day survival (RR 2.31; CI 95% 2.02–2.64) compared to a non-shockable rhythm (39). In this registry analysis, only 16% of the patients with a non-shockable rhythm survived until 30 days (39). In contrast, in our analysis, the corresponding risk ratio for 30-day survival was 1.2 (95%-CI: 0.87–1.68) with an initial shockable rhythm. In our cohort, 30-day survival was higher than reported by Stankovic et al. (39) and significantly higher than reported in the registry analysis (22). Unfortunately, all studies (22, 38, 39) did not report the neurological outcome of the patients.

Furthermore, we can add that most survivors had favorable neurological outcomes. We hypothesize that this resulted from early resuscitative interventions based on mandatory high-quality education spaced over time for the entire anesthesia team. The health-related quality of life was in line with the findings of another in-hospital cardiac arrest cohort outside the operating room from our research group (8).

Compared to cardiac arrest registry data, our study showed real-life data on intraoperative cardiac arrests derived from a large Swiss University Hospital, including cardiac and vascular surgery, an adjunct emergency room for all ages, a cardiac arrest center, and intensive care units.

### 4.1. Limitations

Our study has several limitations. The retrospective design might lead to an underestimation of cases and missing data. Given the overall low number of cases, the statistical analysis, especially for the pediatric cohort, should be seen as descriptive rather than explorative. Unfortunately, the cohort was not assessed by CPC and the SF-12 before hospital admission. The number of assessable survivors for the neurological follow-up might have been higher if they had been contacted directly after 3 months, and the SF-12 could have been more precise. The single-center design might also contribute to difficult generalizability of the data.

## 5. Conclusion

Intraoperative cardiac arrest is rare but is more likely in older patients, patients with ASA physical status ≥IV, cardiac and vascular surgery, and emergency procedures. Patients often present with pulseless electrical activity as the initial rhythm. ROSC can be achieved in most patients. Over half of the patients are alive after 30 days, most with favorable neurological outcomes, if treated immediately.

## Data availability statement

The raw data supporting the conclusions of this article will be made available by the authors, without undue reservation.

## Ethics statement

The studies involving human participants were reviewed and approved by the study protocol was approved by the Ethics Commission of the Canton of Bern (BASEC 2021-02330). According to Swiss Law, participants gave general consent for analyzing their health-related data in the retrospective part of the study and written informed consent for the neurological follow-up. Written informed consent to participate in this study was provided by the participants’ legal guardian/next of kin.

## Author contributions

AF and LF helped with the study design, conduct, patient recruitment, analysis, manuscript writing, and funding. MC-C and MK helped with the study conduct, patient recruitment, and manuscript finalization. ND and UP helped with the study design, analysis, and manuscript finalization. MH helped with the study design, statistical analysis, and manuscript writing. TR and RG helped with the study design, conduct, analysis, manuscript writing, and supervision. All authors contributed to the article and approved the submitted version.

## Funding

This study was funded by a grant from the Burgergemeinde Bern (2022–1029) and the Department of Anaesthesiology and Pain Medicine, Inselspital (FUAD-2-22). The article processing charges were covered by the University of Bern. Open access funding by University of Bern.

## Conflict of interest

RG is the European Resuscitation Council (ERC) Board Director of Guidelines and ILCOR, and the ILCOR Education, Implementation and Team Task Force Chair.

The remaining authors declare that the research was conducted in the absence of any commercial or financial relationships that could be construed as a potential conflict of interest.

## Publisher’s note

All claims expressed in this article are solely those of the authors and do not necessarily represent those of their affiliated organizations, or those of the publisher, the editors and the reviewers. Any product that may be evaluated in this article, or claim that may be made by its manufacturer, is not guaranteed or endorsed by the publisher.

## Abbreviations

ASA: American Society of Anesthesiologists
CPR: cardiopulmonary resuscitation
CPC: clinical performance category
ECPR: extracorporeal cardiopulmonary resuscitation
IHCA: in-hospital cardiac arrest
OR: odds ratio
ROSC: return of spontaneous circulation
SF-12: 12-Item Short Form Survey
