# Supplementary material for: Characteristics and neurological survival following intraoperative cardiac arrest in a Swiss University Hospital: a 7-year retrospective observational cohort study
Source: Front Med (Lausanne). 2023 Jun 15;10:1198078. doi: 10.3389/fmed.2023.1198078 (PMC10309035; doi:10.3389/fmed.2023.1198078)
Supplement: Supplementary file 1 [file Table_1.DOCX]

**Supplementary Table 1.** Baseline characteristics of the patients with intraoperative cardiac arrest stratified according to urgency of the procedure

|  | **All** | **Elective** | **Emergency** | ***p*** | **N** |
| --- | --- | --- | --- | --- | --- |
|  | ***N=195*** | ***N=92*** | ***N=103*** |  |  |
| **Age category:** |  |  |  | 0.258 | 195 |
| Adults (≥16 yrs) | 188 (96.4%) | 87 (94.6%) | 101 (98.1%) |  |  |
| Children (<16 yrs) | 7 (3.59%) | 5 (5.43%) | 2 (1.94%) |  |  |
| **Age** (yrs) | 70.5 [60.0;79.4] | 71.3 [61.3;79.9] | 69.6 [59.7;77.4] | 0.815 | 195 |
| **Sex** (female) | 60 (30.8%) | 30 (32.6%) | 30 (29.1%) | 0.711 | 195 |
| **Height** (cm) | 170 [163;178] | 170 [162;176] | 172 [164;178] | 0.091 | 169 |
| **Weight** (kg) | 75.5 [64.2;89.8] | 74.0 [61.0;88.0] | 77.0 [68.5;90.0] | 0.065 | 178 |
| **BMI** (kg/m^2^) | 26.5 (5.59) | 25.8 (5.14) | 27.2 (6.01) | 0.106 | 168 |
| **ASA physical status:** |  |  |  | <0.001 | 195 |
| I | 1 (0.51%) | 1 (1.09%) | 0 (0.00%) |  |  |
| II | 14 (7.18%) | 13 (14.1%) | 1 (0.97%) |  |  |
| III | 50 (25.6%) | 32 (34.8%) | 18 (17.5%) |  |  |
| IV | 83 (42.6%) | 44 (47.8%) | 39 (37.9%) |  |  |
| V | 47 (24.1%) | 2 (2.17%) | 45 (43.7%) |  |  |
| **Pre-existing illness** (Yes) | 181 (94.3%) | 89 (96.7%) | 92 (92.0%) | 0.271 | 192 |
| **Pre-existing illness category**^1^ |  |  |  |  |  |
| Cardiovascular | 149 (76.4%) | 70 (76.1%) | 79 (76.7%) | >0.99 | 195 |
| Pulmonal | 73 (37.4%) | 34 (37.0%) | 39 (37.9%) | >0.99 | 195 |
| Neurological | 46 (23.6%) | 27 (29.3%) | 19 (18.4%) | 0.105 | 195 |
| Renal | 76 (39.0%) | 36 (39.1%) | 40 (38.8%) | >0.99 | 195 |
| Cancer | 49 (25.1%) | 30 (32.6%) | 19 (18.4%) | 0.035 | 195 |
| Pregnancy | 1 (0.51%) | 1 (1.09%) | 0 (0.00%) | 0.472 | 195 |
| Other^2^ | 33 (16.9%) | 13 (14.1%) | 20 (19.4%) | 0.429 | 195 |
| **Pre-existing condition**^1^ |  |  |  |  |  |
| No | 96 (49.2%) | 54 (58.7%) | 42 (40.8%) | 0.019 | 195 |
| Yes |  |  |  |  |  |
| Sepsis | 17 (8.72%) | 2 (2.17%) | 15 (14.6%) | 0.005 | 195 |
| Hypotension | 31 (15.9%) | 3 (3.26%) | 28 (27.2%) | <0.001 | 195 |
| Metastatic / hematological malignancy | 18 (9.23%) | 8 (8.70%) | 10 (9.71%) | >0.99 | 195 |
| Hepatic / renal insufficiency | 72 (36.9%) | 34 (37.0%) | 38 (36.9%) | >0.99 | 195 |
| **Anesthesia procedure:** |  |  |  | <0.001 | 195 |
| Regional | 3 (1.5%) | 2 (2.3%) | 1 (1.0%) |  |  |
| General and Combined Anesthesia | 180 (92.3%) | 78 (84.7%) | 102 (99.0%) |  |  |
| Monitored Anesthesia Care | 12 (6.2%) | 12 (13.0%) | 0 (0.00%) |  |  |
| **Surgical Intervention:** |  |  |  | 0.001 | 195 |
| open | 165 (84.6%) | 69 (75.0%) | 96 (93.2%) |  |  |
| endovascular | 30 (15.4%) | 23 (25.0%) | 7 (6.80%) |  |  |
| **Type of surgery** |  |  |  | 0.037 | 195 |
| Non-cardiac | 124 (63.6%) | 51 (55.4%) | 73 (70.9%) |  |  |
| Cardiac and vascular | 71 (36.4%) | 41 (44.6%) | 30 (29.1%) |  |  |

Abbreviations: ASA; American Society of Anesthesiologist, BMI; Body Mass Index

^1^a patient could have more than one pre-existing illness or condition, ^2^contained but not limited metabolic, psychiatric, liver and hematological disorders
