# Supplementary material for: Characteristics and neurological survival following intraoperative cardiac arrest in a Swiss University Hospital: a 7-year retrospective observational cohort study
Source: Front Med (Lausanne). 2023 Jun 15;10:1198078. doi: 10.3389/fmed.2023.1198078 (PMC10309035; doi:10.3389/fmed.2023.1198078)
Supplement: Supplementary file 2 [file Table_2.DOCX]

**Supplementary Table 2**. Intraoperative cardiac arrest related data stratified according to the urgency of the procedure

|  | **All** | **Elective** | **Emergency** | ***p*** | **N** |
| --- | --- | --- | --- | --- | --- |
|  | ***N=195*** | ***N=92*** | ***N=103*** |  |  |
| **Time of day** |  |  |  | <0.001 | 195 |
| Daytime (7:00-17:00h) | 133 (68.2%) | 84 (91.3%) | 49 (47.6%) |  |  |
| Nighttime | 62 (31.8%) | 8 (8.70%) | 54 (52.4%) |  |  |
| **Time to cardiac arrest after start of anesthesia** (min) | 109 [41.0;224] | 152 [53.0;272] | 67.5 [35.8;178] | 0.001 | 193 |
| **Initial rhythm during cardiac arrest** |  |  |  | 0.066 | 194 |
| *Shockable* | 45 (23.2%) | 20 (21.7%) | 25 (24.5%) |  |  |
| Ventricular Fibrillation | 28 (14.4%) | 13 (14.1%) | 15 (14.7%) |  |  |
| pulseless Ventricular Tachycardia | 15 (7.73%) | 7 (7.61%) | 8 (7.84%) |  |  |
| Shockable, not further specified | 2 (1.03%) | 0 (0.00%) | 2 (1.96%) |  |  |
| *Non-shockable* | 149 (76.8%) | 72 (78.3%) | 77 (75.5%) |  |  |
| Pulseless electrical activity | 91 (46.9%) | 37 (40.2%) | 54 (52.9%) |  |  |
| Asystole | 24 (12.4%) | 18 (19.6%) | 6 (5.88%) |  |  |
| Bradycardia | 2 (1.03%) | 1 (1.09%) | 1 (0.98%) |  |  |
| Non-shockable, not further specified | 32 (16.5%) | 16 (17.4%) | 16 (15.7%) |  |  |
| **Reasons for cardiac arrest:** |  |  |  |  |  |
| Tamponade (cardiac) | 14 (7.18%) | 5 (5.43%) | 9 (8.74%) | 0.539 | 195 |
| Intoxication | 1 (0.51%) | 0 (0.00%) | 1 (0.97%) | 1.000 | 195 |
| Tension pneumothorax | 1 (0.51%) | 1 (1.09%) | 0 (0.00%) | 0.472 | 195 |
| Hypoxia | 12 (6.15%) | 6 (6.52%) | 6 (5.83%) | 1.000 | 195 |
| Hypovolemia | 54 (27.7%) | 20 (21.7%) | 34 (33.0%) | 0.111 | 195 |
| Hypothermia | 0 (0%) | 0 (0%) | 0 (0%) | . | 195 |
| Hypo- Hyperpotassemia | 8 (4.10%) | 2 (2.17%) | 6 (5.83%) | 0.285 | 195 |
| Hypoglycemia | 195 (100%) | 92 (100%) | 103 (100%) | . | 195 |
| Thrombosis (pulmonary) | 5 (2.56%) | 3 (3.26%) | 2 (1.94%) | 0.668 | 195 |
| Thrombosis (coronary) | 4 (2.05%) | 4 (4.35%) | 0 (0.00%) | 0.048 | 195 |
| Hydrogen ion (acidosis) | 6 (3.08%) | 1 (1.09%) | 5 (4.85%) | 0.216 | 195 |
| Unknown | 47 (24.1%) | 20 (21.7%) | 27 (26.2%) | 0.574 | 195 |
| Other | 73 (37.4%) | 44 (47.8%) | 29 (28.2%) | 0.007 | 195 |
| **Duration CPR until ROSC** (min) | 5.00 [2.00;14.0] | 5.00 [1.00;11.0] | 9.00 [2.50;15.0] | 0.055 | 192 |
| **Defibrillation during CPR** (Yes) | 64 (32.8%) | 33 (35.9%) | 31 (30.1%) | 0.481 | 195 |
| **Number of shocks given** |  |  |  |  |  |
| 1 | 59 (30.3%) | 31 (33.7%) | 28 (27.2%) | 0.405 | 195 |
| 2 | 33 (16.9%) | 18 (19.6%) | 15 (14.6%) | 0.460 | 195 |
| 3 | 17 (8.72%) | 9 (9.78%) | 8 (7.77%) | 0.807 | 195 |
| 4 | 8 (4.10%) | 4 (4.35%) | 4 (3.88%) | 1.000 | 195 |
| 5 | 4 (2.05%) | 2 (2.17%) | 2 (1.94%) | 1.000 | 195 |
| 6 | 3 (1.54%) | 2 (2.17%) | 1 (0.97%) | 0.603 | 195 |
| 7 | 4 (2.05%) | 2 (2.17%) | 2 (1.94%) | 1.000 | 195 |
| **Medication peri-arrest** |  |  |  |  |  |
| Epinephrine | 167 (85.6%) | 73 (79.3%) | 94 (91.3%) | 0.030 | 195 |
| Norepinephrine | 116 (59.5%) | 49 (53.3%) | 67 (65.0%) | 0.127 | 195 |
| Amiodarone | 24 (12.3%) | 14 (15.2%) | 10 (9.71%) | 0.342 | 195 |
| Lidocaine | 4 (2.05%) | 1 (1.09%) | 3 (2.91%) | 0.624 | 195 |
| Vasopressin | 13 (6.67%) | 1 (1.09%) | 12 (11.7%) | 0.008 | 195 |
| Atropine | 21 (10.8%) | 13 (14.1%) | 8 (7.77%) | 0.230 | 195 |
| Bicarbonate | 27 (13.8%) | 12 (13.0%) | 15 (14.6%) | 0.921 | 195 |
| Calcium | 43 (22.1%) | 15 (16.3%) | 28 (27.2%) | 0.098 | 195 |
| Magnesium | 13 (6.67%) | 7 (7.61%) | 6 (5.83%) | 0.833 | 195 |
| **Additional actions during CPR** |  |  |  |  |  |
| (Arterial) Blood test | 66 (33.8%) | 33 (35.9%) | 33 (32.0%) | 0.680 | 195 |
| Pericardiocentesis | 15 (7.69%) | 5 (5.43%) | 10 (9.71%) | 0.396 | 195 |
| Chest tube | 1 (0.51%) | 1 (1.09%) | 0 (0.00%) | 0.472 | 195 |
| Transesophageal Echocardiography | 69 (35.4%) | 31 (33.7%) | 38 (36.9%) | 0.752 | 195 |
| Aortic clamping | 19 (9.74%) | 7 (7.61%) | 12 (11.7%) | 0.479 | 195 |
| RBC Transfusion | 74 (37.9%) | 25 (27.2%) | 49 (47.6%) | 0.005 | 195 |
| FFP Transfusion | 18 (9.23%) | 6 (6.52%) | 12 (11.7%) | 0.323 | 195 |

Abbreviations: CPR; cardiopulmonary resuscitation, ROSC; return of spontaneous circulation, RBC; red blood cell; FFP; fresh frozen plasma
