# Supplementary material for: Characteristics and neurological survival following intraoperative cardiac arrest in a Swiss University Hospital: a 7-year retrospective observational cohort study
Source: Front Med (Lausanne). 2023 Jun 15;10:1198078. doi: 10.3389/fmed.2023.1198078 (PMC10309035; doi:10.3389/fmed.2023.1198078)
Supplement: Supplementary file 3 [file Table_3.DOCX]

**Supplementary Table 3.** Baseline characteristics of the patients with intraoperative cardiac arrest stratified according to 30-day survival

|  | **All** | **Deceased 30-day** | **Survived 30-day** | **p** | **N** |
| --- | --- | --- | --- | --- | --- |
|  | ***N=195*** | ***N=84*** | ***N=111*** |  |  |
| **Age category:** |  |  |  | 0.701 | 195 |
| Adults (≥16 yrs) | 188 (96.4%) | 82 (97.6%) | 106 (95.5%) |  |  |
| Children (<16 yrs) | 7 (3.59%) | 2 (2.38%) | 5 (4.50%) |  |  |
| **Age** (yrs) | 70.5 [60.0;79.4] | 74.1 [62.2;81.8] | 67.2 [57.3;77.1] | 0.024 | 195 |
| **Sex** (female) | 60 (30.8%) | 29 (34.5%) | 31 (27.9%) | 0.406 | 195 |
| **Height** (cm) | 170 [163;178] | 170 [164;176] | 172 [163;178] | 0.822 | 169 |
| **Weight** (kg) | 75.5 [64.2;89.8] | 75.0 [61.0;88.0] | 76.0 [66.0;90.0] | 0.371 | 178 |
| **BMI** (kg/m^2^) | 26.5 (5.59) | 26.0 (6.16) | 26.8 (5.22) | 0.413 | 168 |
| **ASA physical status:** |  |  |  | <0.001 | 195 |
| I | 1 (0.51%) | 0 (0.00%) | 1 (0.90%) |  |  |
| II | 14 (7.18%) | 2 (2.38%) | 12 (10.8%) |  |  |
| III | 50 (25.6%) | 12 (14.3%) | 38 (34.2%) |  |  |
| IV | 83 (42.6%) | 37 (44.0%) | 46 (41.4%) |  |  |
| V | 47 (24.1%) | 33 (39.3%) | 14 (12.6%) |  |  |
| **Pre-existing illness** (Yes) | 181 (94.3%) | 76 (93.8%) | 105 (94.6%) | >0.99 | 192 |
| **Pre-existing illness**^1^ |  |  |  |  |  |
| Cardiovascular | 149 (76.4%) | 68 (81.0%) | 81 (73.0%) | 0.259 | 195 |
| Pulmonal | 73 (37.4%) | 35 (41.7%) | 38 (34.2%) | 0.361 | 195 |
| Neurological | 46 (23.6%) | 19 (22.6%) | 27 (24.3%) | 0.914 | 195 |
| Renal | 76 (39.0%) | 39 (46.4%) | 37 (33.3%) | 0.088 | 195 |
| Cancer | 49 (25.1%) | 17 (20.2%) | 32 (28.8%) | 0.229 | 195 |
| Pregnancy | 1 (0.51%) | 0 (0.00%) | 1 (0.90%) | >0.99 | 195 |
| Other^2^ | 33 (16.9%) | 17 (20.2%) | 16 (14.4%) | 0.378 | 195 |
| **Pre-existing condition**^1^ |  |  |  |  |  |
| No | 96 (49.2%) | 29 (34.5%) | 67 (60.4%) | 0.001 | 195 |
| Yes |  |  |  |  |  |
| Sepsis | 17 (8.72%) | 12 (14.3%) | 5 (4.50%) | 0.032 | 195 |
| Hypotension | 31 (15.9%) | 24 (28.6%) | 7 (6.31%) | <0.001 | 195 |
| Metastatic / hematological malignancy | 18 (9.23%) | 11 (13.1%) | 7 (6.31%) | 0.170 | 195 |
| Hepatic / renal insufficiency | 72 (36.9%) | 37 (44.0%) | 35 (31.5%) | 0.100 | 195 |
| **Urgency**: |  |  |  | <0.001 | 195 |
| emergency |  |  |  |  |  |
| immediate | 63 (32.3%) | 39 (46.4%) | 24 (21.6%) |  |  |
| within 1-6 hours | 28 (14.4%) | 17 (20.2%) | 11 (9.91%) |  |  |
| within 6-12 hours | 5 (2.56%) | 0 (0.00%) | 5 (4.50%) |  |  |
| within 24 hours | 7 (3.59%) | 1 (1.19%) | 6 (5.41%) |  |  |
| elective | 92 (47.2%) | 27 (32.1%) | 65 (58.6%) |  |  |
| **Anesthesia procedure:** |  |  |  | 0.217 | 195 |
| Regional | 3 (1.5%) | 0 (0.00%) | 3 (2.7%) |  |  |
| General and Combined Anesthesia | 180 (92.3%) | 79 (94.0%) | 101 (91.0%) |  |  |
| Monitored Anesthesia Care | 12 (6.2%) | 5 (6.0%) | 7 (6.3%) |  |  |
| **Surgical Intervention:** |  |  |  | 0.817 | 195 |
| open | 165 (84.6%) | 70 (83.3%) | 95 (85.6%) |  |  |
| endovascular | 30 (15.4%) | 14 (16.7%) | 16 (14.4%) |  |  |
| **Type of surgery** |  |  |  | 0.531 | 195 |
| Non-cardiac | 124 (63.6%) | 56 (66.7%) | 68 (61.3%) |  |  |
| Cardiac and vascular | 71 (36.4%) | 28 (33.3%) | 43 (38.7%) |  |  |

Abbreviations: ASA; American Society of Anesthesiologist, BMI; Body Mass Index

^1^a patient could have more than one pre-existing illness or condition, ^2^contained but not limited metabolic, psychiatric, liver and hematological disorders
