# Supplementary material for: Characteristics and neurological survival following intraoperative cardiac arrest in a Swiss University Hospital: a 7-year retrospective observational cohort study
Source: Front Med (Lausanne). 2023 Jun 15;10:1198078. doi: 10.3389/fmed.2023.1198078 (PMC10309035; doi:10.3389/fmed.2023.1198078)
Supplement: Supplementary file 4 [file Table_4.DOCX]

**Supplementary Table 4**. Intraoperative cardiac arrest related data stratified according to 30-day survival

|  | **All** | **Deceased 30-day** | **Survived 30-day** | ***p*** | **N** |
| --- | --- | --- | --- | --- | --- |
|  | ***N=195*** | ***N=84*** | ***N=111*** |  |  |
| **Time of day** |  |  |  | 0.001 | 195 |
| Daytime (7:00-17:00h) | 133 (68.2%) | 46 (54.8%) | 87 (78.4%) |  |  |
| Nighttime | 62 (31.8%) | 38 (45.2%) | 24 (21.6%) |  |  |
| **Time to cardiac arrest after start of anesthesia** (min) | 109 [41.0;224] | 103 [38.5;216] | 112 [44.5;236] | 0.481 | 193 |
| **Initial rhythm during cardiac arrest** |  |  |  | 0.531 | 194 |
| *Shockable* | 45 (23.2%) | 23 (27.4%) | 22 (20.0%) |  |  |
| Ventricular Fibrillation | 28 (14.4%) | 14 (16.7%) | 14 (12.7%) |  |  |
| pulseless Ventricular Tachycardia | 15 (7.73%) | 8 (9.52%) | 7 (6.36%) |  |  |
| Shockable, not further specified | 2 (1.03%) | 1 (1.19%) | 1 (0.91%) |  |  |
| *Non-shockable* | 149 (76.8%) | 61 (72.6%) | 88 (80.0%) |  |  |
| Pulseless electrical activity | 91 (46.9%) | 42 (50.0%) | 49 (44.5%) |  |  |
| Asystole | 24 (12.4%) | 7 (8.33%) | 17 (15.5%) |  |  |
| Bradycardia | 2 (1.03%) | 0 (0%) | 2 (1.82%) |  |  |
| Non-shockable, not further specified | 32 (16.5%) | 12 (14.3%) | 20 (18.2%) |  |  |
| **Reasons for cardiac arrest:** |  |  |  |  |  |
| Tamponade (cardiac) | 14 (7.18%) | 7 (8.33%) | 7 (6.31%) | 0.793 | 195 |
| Intoxication | 1 (0.51%) | 1 (1.19%) | 0 (0%) | 0.431 | 195 |
| Tension pneumothorax | 1 (0.51%) | 0 (0%) | 1 (0.90%) | >0.99 | 195 |
| Hypoxia | 12 (6.15%) | 8 (9.52%) | 4 (3.60%) | 0.161 | 195 |
| Hypovolemia | 54 (27.7%) | 29 (34.5%) | 25 (22.5%) | 0.090 | 195 |
| Hypothermia | 0 (0%) | 0 (0%) | 0 (0%) | . | 195 |
| Hypo- Hyperpotassemia | 8 (4.10%) | 5 (5.95%) | 3 (2.70%) | 0.294 | 195 |
| Hypoglycemia | 0 (0%) | 0 (0%) | 0 (0%) | . | 195 |
| Thrombosis (pulmonary) | 5 (2.56%) | 1 (1.19%) | 4 (3.60%) | 0.393 | 195 |
| Thrombosis (coronary) | 4 (2.05%) | 1 (1.19%) | 3 (2.70%) | 0.636 | 195 |
| Hydrogen ion (acidosis) | 6 (3.08%) | 6 (7.14%) | 0 (0%) | 0.006 | 195 |
| unknown | 47 (24.1%) | 18 (21.4%) | 29 (26.1%) | 0.555 | 195 |
| other | 73 (37.4%) | 27 (32.1%) | 46 (41.4%) | 0.238 | 195 |
| **Duration CPR until ROSC** (min) | 5.00 [2.00;14.0] | 11.0 [4.25;24.5] | 4.00 [1.50;10.0] | <0.001 | 192 |
| **Defibrillation during CPR** (Yes) | 64 (32.8%) | 34 (40.5%) | 30 (27.0%) | 0.068 | 195 |
| **Number of shocks given** |  |  |  |  |  |
| 1 | 59 (30.3%) | 30 (35.7%) | 29 (26.1%) | 0.198 | 195 |
| 2 | 33 (16.9%) | 19 (22.6%) | 14 (12.6%) | 0.098 | 195 |
| 3 | 17 (8.72%) | 9 (10.7%) | 8 (7.21%) | 0.546 | 195 |
| 4 | 8 (4.10%) | 3 (3.57%) | 5 (4.50%) | >0.9 | 195 |
| 5 | 4 (2.05%) | 2 (2.38%) | 2 (1.80%) | >0.9 | 195 |
| 6 | 3 (1.54%) | 2 (2.38%) | 1 (0.90%) | 0.579 | 195 |
| 7 | 4 (2.05%) | 4 (4.76%) | 0 (0%) | 0.033 | 195 |
| **Medication peri-arrest** |  |  |  |  |  |
| Epinephrine | 167 (85.6%) | 81 (96.4%) | 86 (77.5%) | <0.001 | 195 |
| Norepinephrine | 116 (59.5%) | 59 (70.2%) | 57 (51.4%) | 0.012 | 195 |
| Amiodarone | 24 (12.3%) | 16 (19.0%) | 8 (7.21%) | 0.023 | 195 |
| Lidocaine | 4 (2.05%) | 3 (3.57%) | 1 (0.90%) | 0.317 | 195 |
| Vasopressin | 13 (6.67%) | 9 (10.7%) | 4 (3.60%) | 0.093 | 195 |
| Atropine | 21 (10.8%) | 4 (4.76%) | 17 (15.3%) | 0.034 | 195 |
| Bicarbonat | 27 (13.8%) | 20 (23.8%) | 7 (6.31%) | 0.001 | 195 |
| Calcium | 43 (22.1%) | 28 (33.3%) | 15 (13.5%) | 0.002 | 195 |
| Magnesium | 13 (6.67%) | 6 (7.14%) | 7 (6.31%) | >0.9 | 195 |
| **Additional actions during CPR** |  |  |  |  |  |
| (Arterial) Blood test | 66 (33.8%) | 41 (48.8%) | 25 (22.5%) | <0.001 | 195 |
| Pericardiocentesis | 15 (7.69%) | 8 (9.52%) | 7 (6.31%) | 0.573 | 195 |
| Chest tube | 1 (0.51%) | 0 (0%) | 1 (0.90%) | >0.9 | 195 |
| Transesophageal Echocardiography | 69 (35.4%) | 43 (51.2%) | 26 (23.4%) | <0.001 | 195 |
| Aortic clamping | 19 (9.74%) | 11 (13.1%) | 8 (7.21%) | 0.259 | 195 |
| RBC Transfusion | 74 (37.9%) | 45 (53.6%) | 29 (26.1%) | <0.001 | 195 |
| FFP Transfusion | 18 (9.23%) | 14 (16.7%) | 4 (3.60%) | 0.004 | 195 |

Abbreviations: CPR; cardiopulmonary resuscitation, ROSC; return of spontaneous circulation, RBC; red blood cell; FFP; fresh frozen plasma
